# Less Medication Use in Inpatients With Severe Mental Illness Receiving a Multidisciplinary Lifestyle Enhancing Treatment. The MULTI Study III

**DOI:** 10.3389/fpsyt.2018.00707

**Published:** 2018-12-18

**Authors:** Jeroen Deenik, Diederik E. Tenback, Harold F. van Driel, Erwin C. P. M. Tak, Ingrid J. M. Hendriksen, Peter N. van Harten

**Affiliations:** ^1^GGz Centraal, Amersfoort, Netherlands; ^2^School for Mental Health and Neuroscience, Maastricht University, Maastricht, Netherlands; ^3^Tak Advies en Onderzoek, Leiden, Netherlands; ^4^LivIng Active, Santpoort-Zuid, Netherlands

**Keywords:** medication, antipsychotics, physical activity, hospitalization, severe mental illness, schizophrenia, lifestyle, side effects

## Abstract

Besides having an unhealthy lifestyle contributing to premature mortality, inpatients with severe mental illness (SMI) use high dosages of medication. Previous research has shown improved health after lifestyle improvements in SMI. In addition, we aimed to retrospectively study whether a multidisciplinary lifestyle enhancing treatment (MULTI) was associated with changes in medication use after 18 months, as compared with patients that continued treatment as usual (TAU) and explored mediation by a change in physical activity. We conducted an observational study within a cohort of inpatients with SMI, who received MULTI (*N* = 65) or continued TAU (*N* = 49). Data on their somatic and psychotropic medications were collected, converted into defined daily dose (DDD), and analyzed using linear multilevel regression, correcting for baseline value and differences between groups in age, diagnosis, and illness severity. Compared with TAU, the DDD for psychotropic medication significantly decreased with MULTI (B = −0.55, *P* = 0.02). Changes in total activity did not mediate this association, suggesting that multiple components of MULTI contributed. Corrected between-group analyses for subgroups of medication were not possible due to lack of power and skewed distributions. Within-group data showed a decreased proportion of users as well as median DDD in both groups for almost all medications. In addition to previously reported health improvements after 18 months of MULTI, we observed a significant decrease in dose of psychotropic medication in MULTI compared to TAU. This first study evaluating a wide range of medications indicates a possible effect of lifestyle improvements on medication use in inpatients with SMI. Findings need to be confirmed in future controlled studies, however.

## Introduction

In patients with severe mental illness (SMI), a high prevalence of metabolic risk factors (1–3) contribute to their poor cardiovascular health, largely contributing to a reduced life expectancy of at least 7–20 years compared to the general population (4–6). Besides a sedentary lifestyle (7–10), side effects of psychotropic medication (such as weight gain, dyslipidemia, diabetes mellitus, and direct and indirect harm to the vascular system) are associated with these cardiovascular health issues (11–14). Polypharmacy, higher dosages, and longer-term use are associated with higher risk for most of these side effects and somatic diseases (11, 12, 15). A study in people diagnosed with schizophrenia showed that both no exposure as well as high exposure to antipsychotics were associated with higher cardiovascular mortality than either low or moderate exposure (16). Unfortunately, polypharmacy is very prevalent among people with SMI (15, 17) and the dosage of antipsychotic medication has only increased during the last decades for long-term hospitalized patients (18, 19). Alongside this psychotropic medication, patients use antihypertensive, lipid-lowering, antihyperglycemic and other additional drugs if cardiovascular health issues (whether or not caused by psychotropic medication) are too severe (20–24).

Nowadays, there is more focus on the lowest effective dose, on the avoidance of inappropriate polypharmacy to minimize the side effect of antipsychotics (25–28), and on supporting a healthy lifestyle (29–32). Despite an increased number of studies showing benefits of lifestyle interventions in people with SMI, there is a lack of evidence to support the long-term effectiveness of such interventions in hospitalized patients (33, 34). Moreover, to the best of our knowledge, studies evaluating lifestyle interventions in inpatients did not analyze changes in medication use. Recently, improved physical activity and metabolic health were shown after 18 months in this group of patients receiving a multidisciplinary lifestyle enhancing treatment for inpatients with SMI (MULTI) (35). MULTI was pragmatically implemented in a group of inpatients whose psychotropic medication was already critically reviewed prior to its implementation; hence, dose reduction was not a specific goal. The treatment focused on decreasing sedentary behavior, increasing physical activity, and improving dietary habits in the context of daily treatment.

Because the (side) effects of medications are associated with cardiometabolic health issues, we hypothesized that we might observe changes in medication use after lifestyle changes, in addition to improvements in physical health. Therefore, the present study aimed to retrospectively study whether 18 months of MULTI was associated with changes in medication use, as compared with patients that continued treatment as usual (TAU) and to explore whether changes were mediated by increased physical activity as an essential component of MULTI.

## Materials and Methods

### Study Design

This observational cohort study was conducted at wards for long-term inpatient mental healthcare in a psychiatric hospital of GGz Centraal (The Netherlands). The current study is part of a comprehensive evaluation of MULTI, a treatment that was implemented pragmatically in February 2014 in three wards. MULTI was developed because of the identified need to address the comorbidity (e.g., sleep apnea and cardiovascular morbidity) in this population, in part due to a lack of physical activity, obesity, and dietary risks). The implementation of MULTI (described in the next paragraph) was conducted by psychiatrists, nurses and team leaders in collaboration with activity coordinators and a dietitian. During a previous MULTI study (35), data of baseline (Aug.–Dec. 2013) and one follow-up (Aug.–Dec. 2015) were collected from 114 patients (65 received MULTI and 49 continued TAU). Data on types and dose of medication were extracted from electronic patient records and compared between patients receiving MULTI and those receiving TAU. Because of the observational nature of this study, whereby MULTI was already implemented pragmatically in three wards before the start of this study, no randomization took place. Therefore, we analyzed potential differences between groups at baseline and, if significant, corrected for these differences in analyses as potential confounders. The study protocol was approved by the Medical Ethical Committee of the Isala Academy (case 14.0678). All subjects gave written informed consent in accordance with the Declaration of Helsinki.

### Study Population

The cohort consisted of patients with SMI who had been hospitalized for at least 1 year. Patients were included in the MULTI study if they had not received any other intervention related to lifestyle within 18 months since the start of MULTI and if baseline accelerometer data was available. Exclusion criterion was a lack of data after 18 months due to being either discharged or deceased. Patients included for follow-up were dropped out for further analyses if they had insufficient accelerometer data (see Physical Activity below) or refused the repeated accelerometer measurement.

### MULTI

The purpose of MULTI was a holistic lifestyle change with a focus on decreasing sedentary behavior, increasing physical activity, and improving dietary habits. Improving the daily structure formed the base of the treatment, by starting each day with getting up on time, having three joint meals per day, and an active day program consisting of sports-related activities (e.g., walking, running, yoga, biking, indoor team sports), work-related activities (e.g., gardening and helping out with daily jobs), psycho-education (e.g., about side effects, dietary habits), and skills training (e.g., making a grocery list, shopping, cooking). Also, existing policies were reviewed critically—e.g., the use of personal transport within walking distance around the hospital area for every patient was reduced to its use for immobile patients and in case of extreme weather only. Because of heterogeneity in illness severity and different capabilities and interests, the content and intensity of the day-to-day program were tailored to the particular ward and individual patients by the nurses and specific disciplines (see below). Therefore, the actual frequency, intensity, kind of activities and format (e.g., group or alone) could vary between patients and wards. However, it was intended that all patients were doing some of the activities in the morning and afternoon, to prevent prolonged periods lying in bed or sitting at the ward. Also, the participation of nurses in the day-to-day program was an essential element, which contributed to the culture change within the institution and in the provision of support to patients. MULTI was based on a “change from within-principle” developed by current staff (psychiatrists, nurses, and team leader in collaboration with activity coordinators and a dietitian), working with regular context and resources in daily routine care. It was supervised and disseminated per ward by the head practitioner (a psychiatrist) as an innovative treatment method. Nurses received support from the psychiatrists (psycho-education), activity coordinators, and the dietitian. Adherence to and compliance with the treatment was discussed in the weekly multidisciplinary consultation. If a patient could not get along in the day-to-day program (e.g., getting out of bed or attending selected activities), it was agreed upon that specific action was to be taken to physically activate a particular patient, using extra individual motivational interviewing by their mentor (one of the nurses) or psychiatrist, who were trained in this, and by consulting an activity coordinator or dietitian if needed.

Patients who received TAU continued their treatment, which mainly concerned pharmacological treatment and a less structured day program and did not include any supported lifestyle interventions or adjustments.

### Measurements

#### Medication

Medication use was classified by type of medication in the main groups and by converting doses to daily defined doses (DDD) according to the Anatomical Therapeutic Chemical (ATC) Classification System of the World Health Organization (36). Somatic medications included medications for the alimentary tract and metabolism (A), blood and blood-forming organs (B), cardiovascular system (C), and respiratory system (R). Psychotropic medications included the medications for the nervous system (N). Due to the differences in specific purpose, efficacy, and side effects of psycholeptics (N05), we distinguished antipsychotics (N05A) from anxiolytics, hypnotics, and sedatives (N05B & N05C). Likewise, we distinguished the category antipsychotics into three groups: (i) typical and (ii) atypical antipsychotics (occurring in different subgroups) and (iii) lithium (N05AN). For this study, we only used fixed prescriptions (i.e., not including medication to be given as needed). Physical activity.

#### Physical Activity

To test for potential mediation by a change in physical activity, we used accelerometer data (ActiGraph GT3X+) that was collected within the MULTI study. Detailed procedures and settings used in the baseline and follow-up measurement were described elsewhere (9, 35). Accelerometers were worn on the right hip; wear time of ≥6 h/day for ≥3 days was used as the criterion for sufficient measurement, and the same timeframe (9:00 a.m−10:00 p.m) was used for each dataset to be able to compare individual data. These were analyzed using the ActiGraph (*ActiGraph Corp., Pensacola FL, USA*) software ActiLife 6.8.0 and calculated into average total activity counts per hour (TAC/h) as a continuous and detailed outcome variable, where more counts indicate a higher level of activity. The GT3X+ showed a high inter- and intra-instrumental reliability and validity in healthy adults. Inter-instrument reliability in free-living conditions (hip worn) was high for overall activity (ICC = 0.97 and a 95% limit of agreement of ±81.3 counts per minute for vector magnitude) (37, 38) and moderate-to-vigorous activity (ICC = 0.99) (39). The GT3X+ showed acceptable agreement between step counts observed in a laboratory setting (ICC = 0.61–0.99) and free-living situations (ICC = 0.90) (40).

#### Statistical Analysis

Data analyses were performed using SPSS 22.0 and interpreted at a two-tailed significance level of *p* < 0.05. Differences in patient and disease characteristics between patients who received MULTI and those receiving TAU were analyzed using independent *t*-test and chi-square statistics. Continuous variables were examined for linearity, normality, and homogeneity as assumptions for linear analysis by comparing means with medians and analyzing frequency histograms, normality plots, and plots of residuals vs. predicted values. If variables were not distributed linearly toward the dependent variables, they were added as tertiles in the analysis, with the first tertile as the reference category. If data was not distributed normally, analyses were performed using non-parametric tests.

Sum scores of changes in DDDs of somatic and psychotropic medications were distributed normally, after excluding two outliers (*z* < −3) from the DDD of somatic medication. We used linear multilevel regression to evaluate changes in these outcomes between MULTI and TAU, whereby possible clustering of data within wards was taken into account using a two-level structure, with the wards as the first level and the patients as the second. The change scores of the outcome variables were regressed on the treatment variable and adjusted for the baseline value to prevent potential regression to the mean (crude, model 1). We added patient and disease characteristics that significantly differed between patients receiving MULTI and TAU as covariates in models 2 and 3, respectively. We were not able to properly run corrected between-group analyses on subgroups of medication due to skewed distributions and insufficient power for non-parametric between-group analyses. Therefore, we analyzed the change in DDDs within subgroups of medication separately within MULTI and TAU, using Wilcoxon signed-rank tests.

**Figure 1:**
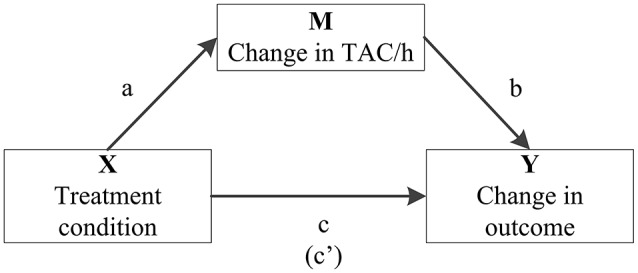
Model summarizing how the role of change in average total activity counts per hour (TAC/h) as a potential mediator (M) in the association between treatment condition (X) and significant change in medication use (Y) is quantified in mediation analysis. It includes the associations between treatment condition and TAC/h (a), TAC/h and the particular outcome variable (b) and treatment condition on the particular outcome variable (c). The coefficient between the latter two, controlling for change in TAC/h, is in parentheses (c′).

Additionally, final significant models were analyzed for possible mediation by a change in total activity counts per hour (TAC/h), using the PROCESS tool in SPSS (41) (Figure 1). The mediation effect was calculated as the product of those coefficients (ab, not shown within the model) and was considered significant if the bias-corrected and accelerated (BCa) bootstrapped confidence intervals did not include zero.

## Results

Table 1 shows the baseline characteristics of patients receiving MULTI and TAU. None of the included 114 patients dropped out. On average, patients receiving MULTI were younger (*M* = −6.45 years, 95% CI: −10.27 to −2.64), had a higher baseline illness severity (*M* = 0.63, 95% CI: 0.17 to 1.08) and were more frequently diagnosed with schizophrenia or other psychotic disorders (*X*^2^ = 21.98, *p* < 0.001) than patients receiving TAU.

**Table 1:** Baseline characteristics of patients (*N* = 114).

**Outcome (scale)**	**MULTI (*n* = 65)**	**TAU (*n* = 49)**
Sex, *n* (%) male	43 (66.2)	27 (55.1)
Age, years, mean (*SD*)	**52.2(8.9)**	**58.7 (11.6)**
Diagnosis, *n* (%)		
Schizophrenia and other psychotic disorders	**61**^a^**(93.8)**	**28**^b^**(57.1)**
Other disorders	**4**^c^ **(6.2)**	**21**^d^ **(42.9)**
Illness severity, CGI-S scale 1-7, mean (*SD*)	**5.0 (1.2)**	**4.3(1.2)**
Years of hospitalization, mean (*SD*)	14.4 (10.9)	13.2 (12.7)

*Significant differences between groups are shown in bold*.

*MULTI, MUltidisciplinary Lifestyle enhancing Treatment for Inpatients with severe mental illness; TAU, Treatment As Usual; CGI-S, Clinical Global Impression–Severity scale*.

a *other: schizoaffective disorder (n = 4) and a psychotic disorder not otherwise specified (n = 1)*.

b *other: schizoaffective disorder (n = 4) and psychotic disorder not otherwise specified (n = 2)*.

c *mood disorders (n = 2): a pervasive disorder not otherwise specified (n = 1) and an anxiety disorder (n = 1)*.

d *mood disorders (n = 8): personality disorders (n = 4), alcohol-related disorders (n = 4), somatoform disorders (n = 2), delirium, dementia, and amnestic and other cognitive disorders (n = 2) and a pervasive disorder not otherwise specified (n = 1)*.

### Total Use of Somatic and Psychotropic Medication

Table 2 shows the number of patients using ≥1 medicine within the relevant groups at baseline and follow-up, including their median DDD and the result of analyzing dose changes within both groups. In both MULTI and TAU, there were fewer patients using somatic medication after 18 months, and their median DDD significantly decreased. The same was observed in the totals for psychotropic medication. Table 3 shows the linear regression estimating the effect of MULTI on the change in DDD sum scores for both somatic and psychotropic medication. After adjusting for baseline sum scores, age, diagnosis, and baseline illness severity, the association with the change in the dose of psychotropic medication remained significant in favor of MULTI. This association was not mediated by a change in TAC/h, as no indirect effect was found (ab = 0.06, 95% BCa CI: −0.01–0.26).

**Table 2:** Number of patients using at least one medicine within main groups of medication at baseline and follow-up, their median daily defined dose, physical activity scores and the results of analyzing changes in dose and physical activity within both MULTI and TAU (*N* = 114).

**Outcome**	**MULTI (*****n*** **=** **65)**									**TAU (*****n*** **=** **49)**								
	**Baseline**				**Follow-up**					**Baseline**				**Follow-up**				
	***n***	**(%)**			***n***	**(%)**			***p***	***n***	**(%)**			***n***	**(%)**			***p***
**Somatic medication**^a^	60	(92)	**2.65**	**(1.51–5.13)**	54	(83)	**0.58**	**(0.50-1.69)**	**<0.01**	48	(98)	**2.35**	**(0.84–3.90)**	42	(86)	**1.00**	**(0.50–2.37)**	**<0.01**
Alimentary tract and metabolism (A)	53	(82)	**2.33**	**(1.38–3.61)**	53	(82)	**0.50**	**(0.50–1.05)**	**<0.01**	46	(94)	**1.83**	**(0.50–3.05)**	40	(82)	**0.50**	**(0.50–1.19)**	**<0.01**
Blood and blood forming organs (B)	2	(3)	3.00	(2.00–^*^)	2	(3)	1.62	(1.00–^*^)	0.72	10	(20)	**1.25**	**(1.00–2.25)**	6	(12)	**0.67**	**(0.30–1.20)**	**0.04**
Cardiovascular system (C)	34	(52)	**0.85**	**(0.36–1.67)**	13	(20)	**0.73**	**(0.33–1.17)**	**<0.01**	24	(49)	**0.42**	**(0.19–1.25)**	12	(25)	**0.83**	**(0.42–1.13)**	**0.02**
Respiratory system (R)	15	(23)	**1.00**	**(1.00–1.50)**	5	(8)	**0.50**	**(0.25–0.75)**	**<0.01**	6	(12)	1.10	(0.92–3.03)	5	(10)	1.00	(0.25–1.35)	0.16
**Psychotropic medication**^a^	65	(100)	**3.74**	**(2.32–5.87)**	49	(75)	**0.79**	**(0.43–1.50)**	**<0.01**	45	(92)	**2.61**	**(1.92–4.49)**	39	(80)	**0.95**	**(0.42–1.83)**	**<0.01**
Antiepileptics (N03)	20	(31)	**0.82**	**(0.38–1.30)**	9	(14)	**0.17**	**(0.08–0.33)**	**<0.01**	7	(14)	**1.00**	**(0.60–1.67)**	5	(10)	**0.20**	**(0.11–0.30)**	**0.02**
Anti–Parkinson (N04)	20	(31)	**0.40**	**(0.40–0.60)**	5	(8)	**0.20**^†^	^†^	**<0.01**	10	(20)	**0.40**	**(0.20–0.53)**	4	(8)	**0.20**^†^	^†^	**0.01**
Typical antipsychotics (N05A)	30	(46)	**1.25**	**(0.72–2.33)**	17	(26)	**0.83**	**(0.23–1.79)**	**<0.01**	20	(41)	0.73	(0.48–1.13)	13	(27)	0.36	(0.13–1.21)	0.07
Atypical antipsychotics (N05A)	53	(82)	**1.25**	**(1.00–1.85)**	37	(57)	**1.17**	**(0.88–1.70)**	**<0.01**	28	(57)	**1.27**	**(0.88–2.00)**	24	(49)	**1.00**	**(0.14–1.88)**	**<0.01**
Lithium (N05AN)	5	(8)	0.90	(0.56–0.90)	3	(5)	0.68	(0.45–^*^)	0.21	3	(6)	0.45	(0.45–^*^)	2	(4)	0.23^†^	^†^	0.10
Anxiolytics, hypnotics & sedatives (N05B+C)	39	(60)	**1.20**	**(0.70–1.80)**	13	(20)	**0.40**	**(0.40–0.80)**	**<0.01**	31	(63)	**1.20**	**(0.50–2.40)**	20	(41)	**0.40**	**(0.20–1.00)**	**<0.01**
Psychoanaleptics (N06)	28	(43)	**1.50**	**(1.00–2.00)**	9	(14)	**0.38**	**(0.29–0.75)**	**<0.01**	23	(47)	**1.00**	**(1.00–1.50)**	9	(18)	**1.00**	**(0.29–1.00)**	**<0.01**
Other nervous system drugs (N07)	2	(3)	0.88	(0.69–^*^)	2	(3)	0.71	(0.18–^*^)	1.00	1	(2)	0.36^†^	^†^	1	(2)	0.18^†^	^†^	0.32
**Physical activity^b^**																		
Wear time during measurement (hours)			55.9	(7.8)			53.6	(9.1)	0.07			55.1	(10.2)			53.3	(10.0)	0.29
Average total activity counts per hour			**29102**	**(12371)**			**33020**	**(14453)**	**0.01**			24636	(14210)			23793	(13151)	0.62

*Significant differences within groups are shown in bold*.

*MULTI, MUltidisciplinary Lifestyle enhancing Treatment for Inpatients with severe mental illness; TAU, Treatment As Usual*.

a Median (IQR)

b Mean (SD)

* *not available due to a low number of users and therefore a lack of dispersion*.

† *the daily dosages are constant*.

**Table 3:** Linear regression estimating treatment effects of MULTI compared to treatment as usual on mean daily defined dose (*N* = 114).

**Outcome (ATC code)**	**B**	**(95% CI)**	***p***
Somatic medications (A, B, C, R)^*^			
Model 1	**−0.48**	**(−0.90–** **−0.06)**	**0.025**
Model 2	−0.44	(−0.88–0.01)	0.054
Model 3	−0.38	(−0.87–0.11)	0.130
**Psychotropic medications (N)**			
Model 1	**−0.47**	**(−0.86–** **−0.07)**	**0.022**
Model 2	**−0.48**	**(−0.90–** **−0.07)**	**0.023**
Model 3	**−0.55**	**(−1.00–** **−0.09)**	**0.020**

*Significant results are shown in bold. MULTI, MUltidisciplinary Lifestyle enhancing Treatment for Inpatients with severe mental illness; ATC code, Anatomical Therapeutic Chemical code; A, Alimentary tract and metabolism; B, Blood and blood-forming organs; C, Cardiovascular system; R, Respiratory system; N, Nervous system*.

*Model 1: crude model, corrected for baseline measurement*.

*Model 2: adjusted model, corrected for baseline measurement and age*.

*Model 3: adjusted model, corrected for baseline measurement, age, diagnosis (schizophrenia and other psychotic disorders, yes/no) and illness severity at baseline*.

* *n = 112, as two outliers were excluded*.

### Analyses on Medication Subgroups

The results of analyses of changes in subgroups of medication are shown in Table 2. For almost all somatic medications, a reduction in the percentage of users was shown in both MULTI and TAU, the largest being in the subgroup regarding the cardiovascular system. The DDD concerning the alimentary tract and metabolism significantly decreased within both groups. For MULTI and TAU this concerned significantly decreased doses of drugs for acid-related disorders (A02; *z* = −4.22, *p* < 0.001), constipation (A06; *z* = −4.68, *p* < 0.001), and diabetes (A10; *z* = −2.97, *p* = 0.003). For TAU, decreases in this main group were also reflected in dose reductions in drugs for acid-related disorders (A02; *z* = −4.0, *p* < 0.001) and constipation (A06; *z* = −4.11, *p* < 0.001), but not for diabetes. Although the median dose of cardiovascular system drugs increased within TAU, the percentage of users decreased. A significant decrease in dose was observed in beta-blocking agents in both MULTI (C07; *z* = −4.00, *p* < 0.001) and TAU (C07; *z* = −3.54, *p* < 0.001). Patients receiving MULTI also used a lower dose for disorders of the respiratory system, as shown by a significant decrease of medication for obstructive airway diseases (R03; *z* = −2.56, *p* = 0.01), while TAU showed a decreased use of medication of blood and blood-forming organs, as reflected by lower use of antithrombotic agents (B01; *z* = −2.06, *p* = 0.04).

For psychotropic medication, a reduction in the percentage of users was shown for almost all subgroups as well, the highest decrease being 40% in users of anxiolytics, hypnotics, and sedatives within MULTI. Both groups showed significant reductions in dose of medications such as antiepileptics, anti-Parkinson drugs, atypical antipsychotics, anxiolytics, hypnotics and sedatives, and psychoanaleptics. In contrast to TAU, MULTI also showed a decrease in the dose of typical antipsychotics.

## Discussion

In the current study, we observed a significant dose reduction in psychotropic medications after 18 months of MULTI, compared to TAU. Although the dose reduction in somatic medication was in favor of MULTI too, it was not significant. When these main groups were split into subgroups, there was a lack of power for corrected between-group analyses. However, the first steps in gaining more detailed insight into dose reductions by using within-group analyses showed interesting leads, for example, in the reduction of medication for diabetes and the respiratory system, which is in line with previously reported physical health improvements within MULTI (35). It also showed clearly the remarkable dose reductions in TAU. It is not likely that this is a time effect, as there was no change of policy in the organization regarding the dosage of medication during these 18 months. The reductions might, however, be a disruptive effect caused by a change in medical staff during the 18 months of this study. The psychiatrist responsible for the majority of the patients within TAU retired and was replaced by a younger colleague, who critically reviewed medication policies. Nevertheless, such a critical review had already been done on the wards receiving MULTI prior to its implementation, so dose reduction was not a specific goal within MULTI. Therefore, the significant decrease in the dose of psychotropic medication is even more striking. One explanation could be that this may be caused partly by decreased psychotic symptoms, but earlier results of the MULTI study showed no significant changes in these symptoms, as compared with TAU, after 18 months (35). Another explanation may be that the psychiatrists reduced the dose of medication based on observed improvements in psychosocial functioning in people who received MULTI (42). It is, however, challenging to clarify specific mechanisms with regard to dose reduction, as there are many variables involved (e.g., interactions between different medications; changes in and preferences of the staff; etc.).

Our findings are somewhat in line with the only other study known to us that evaluated medication use after a lifestyle focused program in patients with SMI (43). In that naturalistic study, Højlund et al. found a decrease in the use of antipsychotics in patients, although they found no specific association between these changes and the degree of participation in the intervention they delivered. However, their reported proportions of patients who used psychotropic co-medication (N categories except for N05) showed little change at follow-up, which is not in line with our observations. Differences may be caused by a different population, as their study was conducted in outpatients with a substantially shorter history of illness, who were relatively young and of whom only a minority used psychotropic co-medication. Also, the absence of a comparison group without lifestyle support makes it difficult to compare results. Indicating decreases in medication use after lifestyle improvement is however very relevant for clinical practice. The ideal situation would be to have medication that addresses the symptoms of psychosis with minimal side effects. However, due to a lack of fundamental innovation in psychopharmacology during recent decades, we still have to work with antipsychotics with a range of motor, metabolic, and cognitive side effects (28, 44). Moreover, as there is still an overall favorable benefit-to-risk ratio for the use of antipsychotics (45), addressing lifestyle is of importance to help minimize such side effects. Besides previously reported improvements in physical health (35), current results show that an integrated multidisciplinary approach is associated with a decrease in the dose of medication. Thereby, it potentially reduces (the risk for) those side effects, which are partly dose-dependent (11, 46). Additionally, because many but not all side effects are reversible, it suggests that lifestyle improvements could contribute to the prevention of irreversible effects, e.g., motor side effects. The reductions found are also promising from the patient's point of view, as side effects and negative attitudes toward medication use are associated with non-adherence, distress, and life impact (47–50).

Our explorative mediation analysis showed that the increase in physical activity observed in patients receiving MULTI after 18 months (35) did not mediate the association between MULTI and change in psychotropic medication use in this same period of time. This indicates that the decrease of psychotropic mediation was not a result of just increasing physical activity. We hypothesize that, with an organizational culture change as a base, multiple components that complement physical activity contribute to improvements, including focus on dietary habits, psycho-education, personal tailoring, and support by peers and qualified participating staff, in line with recent studies advocating the use of such elements (29, 30, 51–53). Such a holistic approach, in which patients are encouraged to do any activities instead of none (i.e., decreasing sedentary behavior), may be more feasible and beneficial in the longer term for inpatients with SMI. While previous studies have shown that structured exercise is feasible in those with SMI in general (31), sedentary behavior (as distinct from physical inactivity) is highly prevalent and associated with cardiometabolic risks that are largely independent of time spent in structured exercise (7, 8). Thus, controlled trials are needed to examine further the feasibility and effects of interventions targeting sedentary behavior.

Limitations in this study mainly arise from the naturalistic and observational design. In this design, we were not able to randomize patients (e.g., the TAU wards treated fewer patients with psychotic disorders) and control for treatment settings in advance (e.g., the continuity of psychiatrists). In addition, although dose reduction was not a specific goal of MULTI, psychiatrists were not blinded for patients' treatment condition (i.e., receiving MULTI or not), which might have affected observed dose reductions. Although this observational study seems the first indicating a possible effect of lifestyle improvements on medication use in inpatients with SMI, future well-designed trials are needed to study the efficacy of lifestyle changes on medication use. Regarding the participation of patients in MULTI, we have no specific data on (the degree of) their adherence. It was intended that all patients were doing some activities in the morning and afternoon, tailored to the particular ward and patients' abilities and interests. However, data on adherence to these individualized day-to-day programs including more detailed data on participation (e.g., frequency and intensity of activities) would make it possible to study the association between the (degree of) adherence and treatment outcomes. Also, our analyses depended on the availability of data within the cohort. Nevertheless, by using multilevel analyses adjusting for differences between MULTI and TAU, we aimed for robust results. However, more data will be needed to have sufficient power for adequate between-group analyses within subgroups of medication, whereby we could gain more detailed insight into specific reductions as indicated by within-group analyses (e.g., drugs for diabetes or obstructive airway diseases). This would provide the opportunity to control for possible differences between groups such as age, which can affect the prescribed dosages of medication (54–56). Furthermore, we used a simple mediation model to explore the influence of a change in physical activity, as an essential element of MULTI, on the association between MULTI and change in medication use. However, the main limitation of this is that by measuring all variables within the mediation analyses nearly simultaneously, we assumed that the association between change in total activity and change in medication use between our pre (T1) and post measurement (T2) would be the same as we would have measured between T2 and a (theoretical) third time point. To study if physical activity mediates associations between lifestyle interventions and medication use, in general, it would be the best to measure physical activity as a middle time-point to study the effect of a change in physical activity over time. Finally, we did not include pro necessitate medication (i.e., to be given as needed) because we cannot specify if and to what extent patients took these medications. The strength of this study is that it is the first study to report detailed dose-specific data on changes in both somatic and psychotropic medication use in inpatients with SMI after lifestyle changes. Despite the limitations, first steps were taken to analyze associations between lifestyle improvements and medication use in these patients, who are suffering from severe physical health problems and receive high dosages of medication. The naturalistic setting of the study improves the generalizability of the results and meets the need for observational studies to supplement randomized controlled trials in order to improve the external validity, such that clinicians treating patients in real-world settings have relevant evidence on which to base their clinical decisions (57). Regarding the impact of critically reviewing medication use that was most likely observed in TAU, it is a strength in favor of MULTI that medication was already critically reviewed on these wards before its implementation, so dose reduction was not a specific goal within MULTI. This suggests that lifestyle improvement has added value with regard to decreasing the dose of medication in inpatients with SMI. Above all, this strengthens the fact that we compared it with TAU. Otherwise, we would only have observed the improvements in MULTI without any context. Moreover, by analyzing possible mediation by total activity, we took a first step to gaining more insight into the contribution of different elements of MULTI to the observed improvements. Finally, by including baseline measures in this analysis, our mediation analysis is mathematically in line with a covariance approach, which was the recommended method for mediation analyses (58, 59).

In summary, in addition to previously reported improvements in physical activity and metabolic health after 18 months of MULTI, we observed a significant decrease in dose of psychotropic medication as compared with TAU. We encourage further longitudinal controlled research to gain more insight into the relationship between lifestyle changes and possible dose reductions to improve health outcomes in inpatients with SMI.

## Data Availability Statement

Data are available from the corresponding author on reasonable request and with permission of GGz Centraal.

## Author Contributions

JD: obtaining funding, study design, retrieving, processing and analyzing data, drafting the manuscript; DT: study design, supporting retrieving, processing and analyzing data; HvD: retrieving, processing and supporting analyzing data; IH: supporting analyzing data; DT and PvH: supporting grant applications; All authors critically revised the manuscript and approved the final version.

### Conflict of Interest Statement

The authors declare that the research was conducted in the absence of any commercial or financial relationships that could be construed as a potential conflict of interest.
